# An Asymptomatic and Overelongated Styloid Process

**DOI:** 10.1155/2017/7971595

**Published:** 2017-01-26

**Authors:** Emrah Soylu, Ahmet Altan, Ahmet Ercan Sekerci, Nıhat Akbulut

**Affiliations:** ^1^Faculty of Dentistry, Department of Oral and Maxillofacial Surgery, Gaziosmanpasa University, Tokat, Turkey; ^2^Faculty of Dentistry, Department of Oral and Maxillofacial Radiology, Erciyes University, Kayseri, Turkey

## Abstract

Elongation of the styloid process is a rare condition. Only 4% of patients have clinical symptoms where elongated styloid process (ESP) occasionally irritates or disrupts adjacent anatomical structures, which is called Eagle syndrome. This present report was aimed at reporting an asymptomatic ESP with unusual width and length.

## 1. Introduction

Styloid complex consists of styloid process, styloid ligament, and stylomandibular ligament [1]. Styloid process and ligament are derived from first and second branchial arches and Reichert's cartilage. During fetal development, Reichert's cartilage links to styloid bone to the hyoid bone. Styloid process is derived from the temporal bone just before the stylomastoid foramen. Apex of styloid process is clinically important as it is placed between external and internal carotid arteries [2]. Also, the facial nerve runs anteriorly and medially to the styloid process.

Normal length of styloid process in adults can vary between 20 and 25 millimeters [2]. Styloid processes longer than 30 mm are called elongated styloid processes (ESP) [3–5]. Incidence of elongation of styloid process is around 4–7%; only 4% of patients with elongation of styloid process show the symptoms [3, 6]. In the event that elongation of styloid process causes clinical symptoms like neck and cervicofacial pain, it is described as Eagle syndrome [4, 7]. This sign and symptom are believed to be formed due to styloid process pressure on some nerve and vascular structures situated around the styloid process like facial nerve or internal or external carotid arteries. More occasionally, dysphagia, tinnitus, and otalgia can occur in Eagle syndrome. Medical treatment is the first step for Eagle syndrome. Surgery containing resection of elongation of styloid process is accepted as certain. Elongation of styloid process is diagnosed via radiological and physical examination. Panoramic radiographs are also commonly used for diagnosis of elongation of styloid process.

This article aims to present clinical and radiological symptoms of a unilateral, huge, asymptomatic ESP.

## 2. Case Report

**Figure 1:**
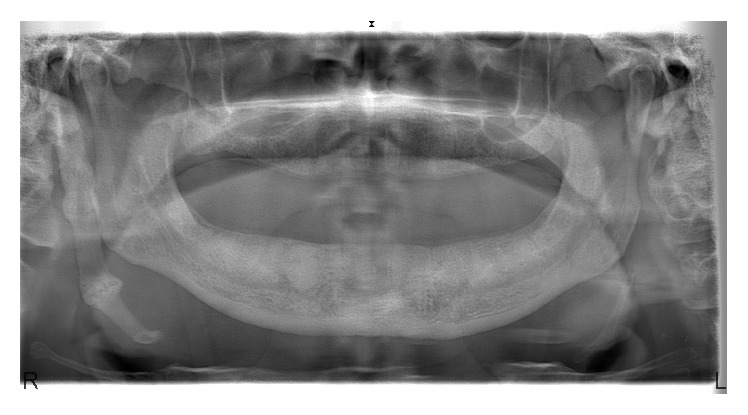
Patient OPG.

A 68-year-old, otherwise healthy, male patient was referred to our clinic with the main complaint of the pain in mandible. His history was free of systemic diseases and patient stated that pain occurs especially when wearing the lower denture. Clinical examination showed irregular mandibular crest with bone spurs and it was noticed that the cause of complaint was the pressure to the irregular bone structures of mandible when the patient wore the lower denture. An Orthopantomograph (OPG) was taken for routine radiological examination. In radiological examination, a bony structure that lies near the right mandibular ramus and reaches submandibular region was noticed incidentally and defined as ESP (Figure 1).

**Figure 2:**
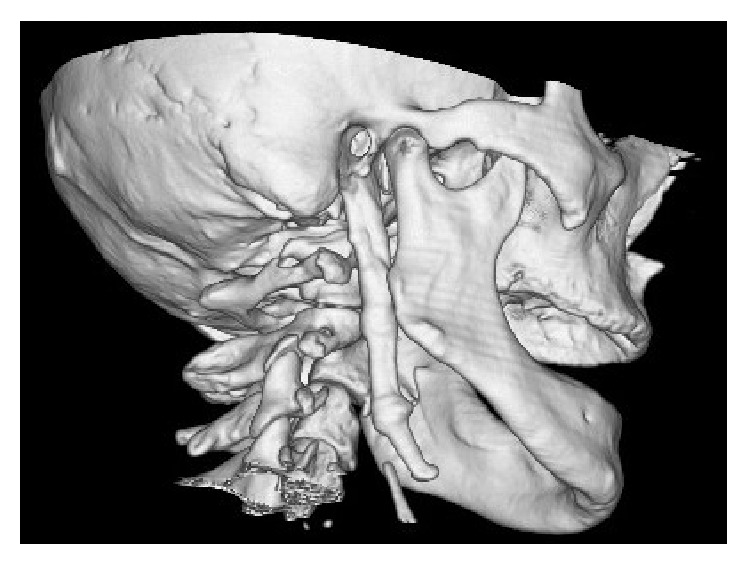
3D reconstruction of the tomographic images. Sagittal view of the ESP.

**Figure 3:**
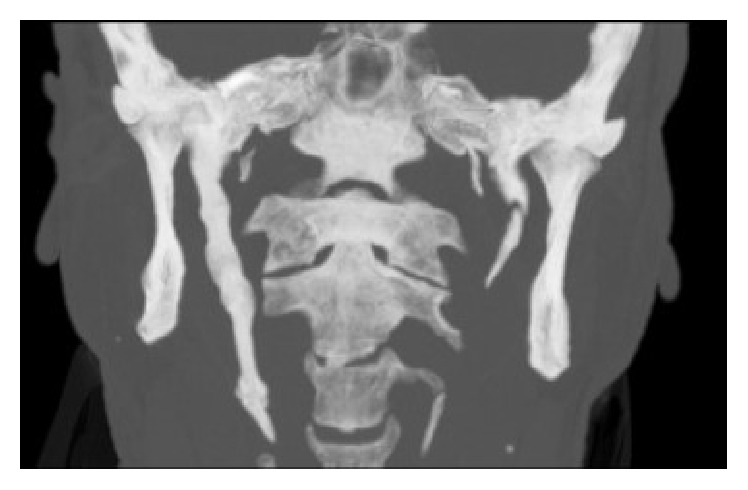
Coronal view of ESP.

Patient was asked for pain during neck movement or neck pain in rest, but he stated that he was free of symptom. Also, the inferior part and tip of ESP can be palpable via submandibular space; however patient was free of pain on palpation. A head and neck tomography was taken and tomographic examination confirmed the ESP diagnosis with a styloid process 80,4 mm in length and 8,3 mm in width (Figures 2 and 3).

Patient was informed about the clinical situation and was told to appear for a routine clinic visit every 6 months.

## 3. Discussion

Elongation of styloid process was described by Eagle for the first time as clinical symptoms and signs seen with structural changes in styloid ligament. There were some studies done using a panoramic radiograph and a three-dimensional computed tomography scan on length of styloid process [8–10]. In their studies, Thot et al. [11] have reported that left styloid process length is between 0.7 and 1.6 centimeters and right styloid process length is between 0.8 and 2.4 centimeters. In the same study, average styloid process length is reported to be 1.49 cm on the right and 1.52 cm on the left [11]. Jung et al. [5] have stated that, in order for the styloid process to be deemed elongated, the length should exceed 45 millimeters. In our case, elongation of styloid process was seen on the unilateral right side. In the measurements taken, styloid process was 80,4 mm in length and 8,3 mm in width. When compared to literature, it can be accepted that the patient has elongation of styloid process.

Panoramic radiographs were seen as an important tool for diagnosis of elongated styloid process [12]. Panoramic radiographs are often preferred as they are routine and simple method to be used in maxillofacial area. Incidence of elongated styloid process varies greatly in population. Incidence is seen to vary between 4% and 28% in studies done with panoramic radiographs [4, 13] and this group had symptoms with a ratio of 4%–10.3% percent [12]. Elongated styloid process was seen in panoramic radiography of a patient who applied to our clinics for in routine dental examination. Styloid process of this patient was big in size and surprisingly asymptomatic.

There were no relations detected between elongation of styloid process and gender [12]. There is no consensus on age between the researches. Some researchers stated that incidence of elongation of styloid process is higher between the ages of 30–40 [3] whereas some state the ratio is higher in individuals older than 50 years [14]. In the studies, it was reported that elongation of styloid process is often seen as bilateral [14, 15]. In the presented case, elongation of styloid process in a 68-year-old male patient is detected as unilateral, in contrast to literature.

As elongated styloid process creates symptoms, it can be treated medically or with surgery. Medical treatment consists of use of steroids, local anesthesia, and oral carbamazepine. But medical treatment does not result well in the long term [16]. Styloid process can be done via intraoral and extraoral approach based on surgery methods. In extraoral approach, a good view is obtained and vascular major complications are inhibited but this operation lasts long and cosmetic satisfaction is low on extraoral scar tissue. Intraoral approach lasts shorter when compared to extraoral approach and inhibits aesthetical problems and less dissection is needed during this procedure [17]. As no symptoms were seen in patients with styloid process of huge size, no medical or surgical treatments were applied. The patient's routine follow-up has continued.
